# Structural and Morselized Allografting Combined with a Cementless Cup for Acetabular Defects in Revision Total Hip Arthroplasty: A 4- to 14-Year Follow-Up

**DOI:** 10.1155/2018/2364269

**Published:** 2018-02-04

**Authors:** Hou-Tsung Chen, Cheng-Ta Wu, Tsan-Wen Huang, Hsin-Nung Shih, Jun-Wen Wang, Mel S. Lee

**Affiliations:** ^1^Department of Orthopaedic Surgery, Kaohsiung Chang Gung Memorial Hospital, Kaohsiung, Taiwan; ^2^Department of Orthopaedic Surgery, Chang Gung Memorial Hospital, Chiayi, Taiwan; ^3^Department of Orthopaedic Surgery, Chang Gung Memorial Hospital, Linkou, Taiwan

## Abstract

Using morselized and structural allograft to restore bone stock for massive acetabular bone defect in revision total hip arthroplasty (THA) is an appealing procedure. However, concerns about inability to achieve long-term stability following allograft resorption remained. From 2003 to 2012, 59 hips in 58 patients undergoing revision THA for Paprosky type II or III acetabular defects were retrospectively reviewed. The acetabular defects were managed with deep-frozen morselized and structural allografts, and a press-fit cementless cup along with supplementary screws. Clinical outcomes and radiographic results were analyzed with a mean follow-up of 8.7 years. The clinical successful rate was 100% for hips with Paprosky type II defect, 95.2% for IIIA defect, and 92.8% for IIIB defect. Three hips with type III defect failed at 4, 7, and 9 years, respectively. Harris Hip Score improved significantly from 60.1 preoperatively to 91.3 at the latest follow-up. All hips with good clinical results showed trabecular bridging in the allograft-host bone interface. Deep-frozen structural and morselized allograft in combination with a press-fit cementless cup represented a viable option to reconstruct acetabular defects in revision THA.

## 1. Introduction

The revision burden for total hip arthroplasty (THA) is increasing worldwide [1]. The surgery is challenging especially when dealing with the bone deficiency caused by multiple surgeries, periprosthetic joint infection, and osteolysis [1–3]. The management of massive acetabular deficiency at revision arthroplasty is amongst the greatest challenges in hip surgery. The cardinal principles of reconstruction of acetabular defect are restoring the bone stock, establishing a solidly fixed prosthesis-bone construct, achieving bony unity, and obtaining durable implants survival.

The severity of the bone deficiency and the quality of remaining host bone on the acetabular side often dictated the ways of reconstruction during revision surgery. For minor cavitary or contained defects, impaction bone grafting by morselized or smaller-sized strut allograft along with a cementless hemispherical component was usually the reconstruction of choice [4]. For major segmental or uncontained acetabular defects, hemispherical revision shells combined with structural allografts, porous tantalum augments, cup-cages, reconstruction cages, or custom triflanged reconstruction ring are often needed [5–8].

Modular porous metal augments have been increasingly popular to cope with segmental bone loss [9]. The advantages of using porous metal augments include easier surgical technique and no risk of disease transmission [10, 11]. In addition, porous metal augments are reliable for bone ingrowth and will not be resorbed [9, 12, 13]. However, they are not biological [13], and this technique may be reserved for those patients in which further revision is not anticipated [9, 13]. As a contrast, allograft reconstruction presents a viable choice for massive acetabular deficiency because it provides a biocompatible scaffold for revascularization, creeping substitution, remodeling, and eventually restoration of the host bone [14]. Excellent results have been reported by using morselized allografting technique with cemented or cementless acetabular component [4, 15]. Although structural allografts were reported to successfully restore the bone stock and make subsequent revision less complicated [13], controversies of using structural allografting remained due to the early failure associated with inadequate initial stability, progressive implant loosening, and allograft absorption [16].

Because using structural allografting in cementless revision THA is less reported in the literature, the study retrospectively reviewed a group of patients undergoing revision for medium to large acetabular defects using morselized and structural allograft in combination with a cementless revision cup followed for 4 to 14 years.

## 2. Materials and Methods

### 2.1. Patients

From January 2003 to December 2012, patients undergoing revision THA by a single surgeon for acetabular bone deficiency were retrospectively reviewed. The study was conducted with a waiver of patient consent but was approved by the Institution Review Board of our Hospital (IRB201700086B0). The inclusion criteria for the retrospective analysis consisted of a minimum follow-up of 4 years and using morselized and structural allograft for acetabular reconstruction, in combination with a cementless hemispherical cup by a press-fit technique and multiple screws fixation. The exclusion criteria were minor acetabular bone loss (Paprosky type I), using morselized allograft alone, pelvic discontinuity, or revision using a cemented cup, porous metal augment, or a reconstruction cage.

A total of 59 hips in 58 patients who met the criteria above were included for analysis. There were 23 male patients (23 hips) and 35 female patients (36 hips). Indications for revision THA included aseptic loosening of acetabular component or reconstruction cage, acetabular wear or bone erosion following hemiarthroplasty, and prior periprosthetic hip joint infection. The management of periprosthetic joint infection followed the standard two-staged protocol with the first-stage resection arthroplasty and implantation of mobile antibiotic-laden cement spacer, and the second-stage reimplantation THA and reconstruction of bone defects [17].

### 2.2. Surgical Technique

Acetabular bone loss was defined and evaluated according to the Paprosky classification based on the findings at the surgery and preoperative radiographs [15]. With the patient in the lateral decubitus position, a direct-lateral transgluteal approach was used. The previous prosthesis or cement spacer was removed. The osteolytic lesions and the hypertrophic tissues in the acetabulum were thoroughly debrided until the remaining host bone was exposed. Care should be taken not to violate the tissues connected to the retroperitoneal or the intrapelvic space. At this point, the bone defects were carefully evaluated and estimated for the amount of allografts needed for reconstruction. Provisional reaming of the acetabulum was performed until punctate bleeding from the healthy host bone was encountered. Autologous grafts obtained during debridement and reaming were carefully preserved. Deep-frozen morselized allografts ranging from 1 mm to 10 mm in size were paved to the floor of the bone defects while structural allografts were impacted to those cavitary defects that are more than 2.5 cm in size. The grafts were solidly impacted and incorporated with the host bone by reverse reaming of a 2-mm smaller acetabular reamer to the size of the press-fit cup. If the reamer could be well engaged with the host bone in the ideal orientation by the reverse reaming, the primary stability of a press-fit cup could usually be achieved. Otherwise, segmental allografts were required to provide additional support either to the acetabular dome or to the column deficiency. The structural allograft was carved and shaped to be fixed onto the periphery of the acetabulum by multiple 4.5 mm cancellous screws. The preserved autologous grafts were impacted to the gap between the structural allograft and the host bone. Reverse reaming was performed again until the reamer could be engaged well with the reconstructed acetabular wall in the ideal orientation. At this point, at least 40%~50% contact to the host bone was assessed. Usually primary stability of a press-fitting titanium cup or trabecular metal cup could be achieved with supplementary screws. If the primary stability could not be obtained, conversion to either cemented cup or reconstruction cage will be done.

### 2.3. Outcome Assessment

Postoperatively, patients were instructed to follow standard precautions and to remain partially weight-bearing for 6 to 12 weeks depending on the clinical and radiographic assessment. The patients were reviewed at 6 weeks and 3, 6, and 12 months after the index surgery and annually thereafter. Standard anteroposterior pelvis and lateral hip radiographs were routinely followed at every visit. Radiographic evaluations were done by 3 senior surgeons who were independent from the review of clinical results. Implant position, radiolucency in DeLee and Charnley zone [18], allograft-prosthesis interface, and host bone-allograft interface were carefully evaluated. Migration of the prostheses was defined according the criteria of Hendricks and Harris [19] and Onsten et al. [20], with a change of cup position by greater than 2 mm horizontally or vertically or change of cup angle by greater than 5 degrees being radiographically meaningful. The radiolucencies at bone-prostheses interface were measured radiographically following the description by DeLee and Charnley [18], and the radiolucent lines greater than 2 mm were considered positive. Conn's method [21] was used to assess the incorporation of the allograft, by which the presence of clearly delineated trabeculae bridging over host-graft junction was defined positive. We judged the graft to be successfully incorporated when the allogenous cancellous structure not only acquired the same radiodensity as that of the supporting pelvic bed but also had a continuous trabecular pattern in between. The functional outcome was assessed by Harris Hip Score [22]. Postoperative complications including periprosthetic joint infection, aseptic implant loosening, or instability were all recorded.

## 3. Results

All 59 hips were available for a mean follow-up of 8.7 years (range, 4 to 14 years). The mean age of the patients at the time of revision was 62.3 years (range, 39 to 84 years). Preoperative diagnoses for revision THA included aseptic loosening of cup in 54 hips (91.5%), aseptic loosening of reconstruction cage in 2 hips (3.3%), acetabular bony erosion following hemiarthroplasty in 2 hips (3.3%), and prior periprosthetic joint sepsis with infection being eradicated in 1 hip (1.9%) (Table 1). Ten hips belonged to Paprosky type II defect (6 with IIA, 3 with IIB, and 1 with IIC) and 49 hips to Paprosky type III defect (21 with IIIA and 28 with IIIB). There were 15 hips that underwent femoral components revision at the same time. The reasons for femoral components revision included one hip with prior periprosthetic joint sepsis, 5 hips with stems malposition, and 9 hips with aseptic femoral component loosening. All the revisions were done by cementless long stems.

At a mean follow-up of 9.1 years, all 10 hips with Paprosky type II defect survived successfully (Table 2). Neither implant migration nor radiolucency in DeLee zone was noted radiographically at the latest follow-up. Trabecular bridging was noted over the interface of the allograft and host bone at a mean of 9.5 months (range, 6 to 16 months). There was no sign of allograft absorption. Mean Harris Hip Score improved from 68.5 (range, 52.1–73.2) preoperatively to 93.3 (range, 87.5–98) postoperatively (*p* value < 0.001).

**Figure 1:**
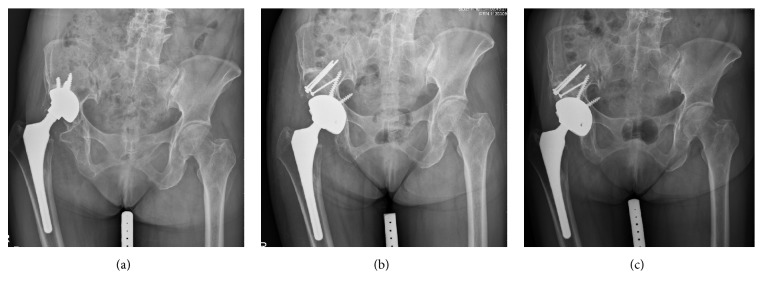
A 61-year-old female patient, with aseptic loosening of right acetabular component. (a) Preoperative radiography showed superolateral cup migration and massive bone defect (Paprosky IIIA). (b) Revision with structural allograft and Trilogy Jumbo cup. (c) Follow-up radiography at 4 years showed stable cup position and good incorporation of allograft.

**Figure 2:**
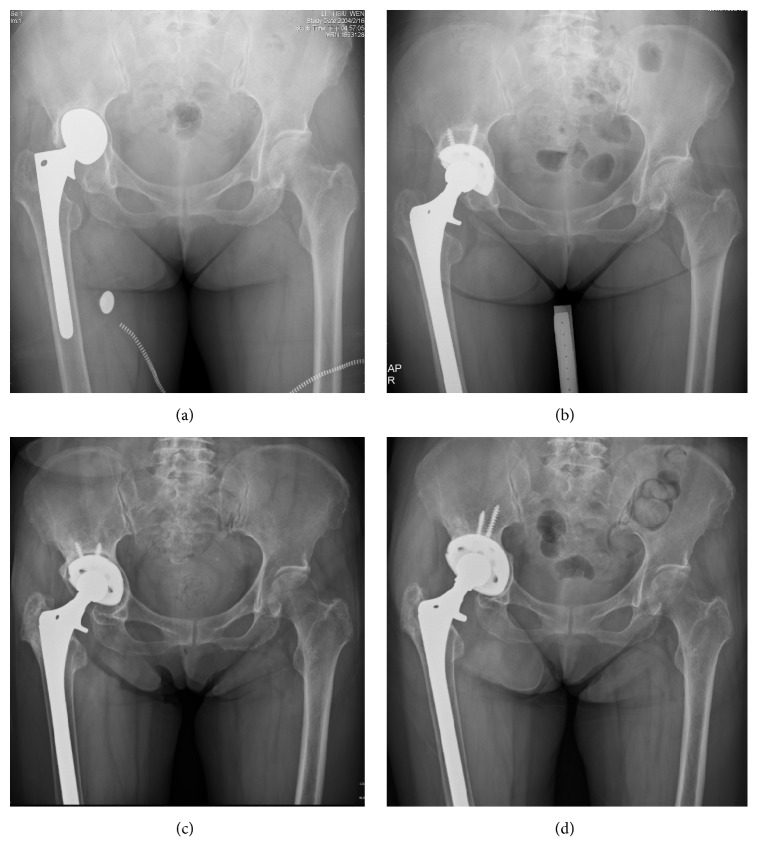
A 69-year-old female patient, with aseptic loosening of right bipolar hemiarthroplasty with acetabuli protrusio. (a) Preoperative radiographs showed up-and-in migration of the bipolar prosthesis with violation of Kohler's line (Paprosky IIIB). (b) The acetabular component was revised with structural and morselized allograft and Trilogy cup. (c) Loosening of the acetabular component at 7 years after 1st revision THA. (d) The 2nd revision was undertaken by allogenous bone grafting again and a titanium porous revision cup. Radiography at 6 years showed stable cup position and good incorporation of allograft.

For Paprosky type III defect, 46 hips (94%) showed trabecular bridging over host-allograft interface at a mean of 13 months (range, 8 to 20 months) (Figure 1). Radiolucent lines in DeLee zones were demonstrated in 4 patients (3 in zone I, 1 in zone II), but all were less than 2 mm and asymptomatic. Mean Harris Hip Score improved from 58.4 (range, 45.2–71.2) preoperatively to 90.8 (range, 80.1–95) postoperatively (*p* value < 0.001). There were 3 hips (1 with type IIIA and 2 with type IIIB) that failed at 4, 7, and 9 years after the index surgery, respectively, because of aseptic loosening over the acetabular components. All 3 patients undertook another revision surgery at the time of failure. During the 2nd revision surgery, the acetabular defects were downgraded to Paprosky type II lesions in all 3 patients because of successful incorporation of prior allograft to the host bone. Two of them used trabecular metal cup with larger diameter for revision, and the other one was managed with allografting again and a press-fitting titanium porous revision cup (Figure 2). All 3 patients had successful clinical outcomes following the 2nd revision surgery.

The overall clinical successful rate of revision THA using the combination of morselized and structural allografting with a cementless cup in the study was 94.9%. The successful rate was 100% for hips with Paprosky type II defect, 95.2% for IIIA defect, and 92.8% for IIIB defect. The mean time for allograft incorporation to the host bone was 12.5 months (range, 6 to 20 months). There was one patient that sustained acute periprosthetic hip joint infection one month after the index revision surgery. The infection had been successfully treated by debridement, antibiotic administration, and implant retention. All of the revised femoral components were fixed well without loosening at the latest follow-up. There was no dislocation or nerve palsy after revision THA in this series.

## 4. Discussion

The increment in the numbers of primary THA is expected to generate a larger numbers of revision procedures, particularly in younger and more active patients [23]. The difficulties in coping with the acetabular bone deficiency are challenging in hip surgery. Surgical options include impaction grafting, structural bone grafting, porous metal augments, and the use of cement with or without acetabular cages and rings. Amongst them the use of bone graft to reconstitute deficient bone stock may still be the most attractive strategy. The current study described favorable short-to-midterm results of using morselized and structural allograft in combination with a press-fit cup to manage Paprosky II and III acetabular defects. The overall successful rate in this series was 94.9% and it was 100% for hips with Paprosky type II defect, 95.2% for IIIA defect, and 92.8% for IIIB defect. The results were comparable and slightly superior to the other reports [24, 25].

The use of impaction bone allograft along with a cemented acetabular component in revision THA was first described by Slooff et al. At a minimum of 10-year follow-up (mean 11.8 years), the survival rate was 94% using aseptic loosening as the endpoint [26]. However, other studies showed unfavorable survivorship using Slooff's cementing allograft technique [27, 28]. The majority of failures were found in type III defect with a combined cavitary and segmental bone loss [29]. In addition, progressive radiolucency in bone-cement mantle and loss of component stability in long-term follow-up were reported [16, 30]. To improve the survivorship, biological fixation using a cementless press-fit cup and allografting has been reported [31]. Evolvement in metallurgy and surface management on acetabular components by porous-coating with sintered-bead technology and titanium mesh significantly improved the ability of biological fixation and long-term durability [32]. Della Valle et al. reported excellent long-term results using allograft and cementless cup in revision THA for failed acetabular component [33]. Palm et al. also demonstrated satisfactory results in acetabular revision with massive allograft impaction and porous-coated cup, with a 90.5% survival rate at a mean of 9 years [34]. In the current study, the use of press-fit cup supplemented with multiple screws in the context of using morselized and structural allograft to restore bone stock also showed promising outcomes.

There are still concerns about early mechanical failure, prolonged allograft incorporation, and bone resorption in the employment of morselized and structural allograft in the management of type III acetabular deficiency [16]. Hooten Jr. et al. reported unsatisfactory results and loosening of acetabular components in that poor vascularization and long-term remodeling of the grafts might lead to their absorption [14]. Garbuz et al. reported only a 55% survival rate at 7 years after major column allograft reconstruction [16]. However, successful outcomes of cementless cup and allografting technique to treat type III defects have also been demonstrated [31–35]. The main benefit of structural allografts was the capacity to restore bone stock, making any subsequent revision less complicated particularly in young patients. Nevertheless, the more the acetabular component was supported by the allograft, the greater the risk of failure noted was [36]. It has been reported that the potential of creeping substitution by the host bone was limited by a surface-to-volume ratio, with proportions of “internal repair” as little as 20% in massive allografts [13, 37]. We thought the successful results in this study could be related to the following factors. (1) Morselized grafts were used to pave all the cavities that also filled up the gap between the structural allografts and the host bone. (2) The morselized and structural grafts were reversely reamed to be solidly incorporated with the host bone. (3) Autologous grafts were used to fill up the gap between the structural allografts and the host bone. The mean time of allograft incorporation was 12.5 months in our series, which was comparable to the range (12–17 months) reported in other studies [38, 39]. (4) At least 40% to 50% of host bone contact of the press-fit cup was obtained. Inability to obtain greater than 40%–50% host bone contact was deemed inappropriate for reconstruction simply by uncemented hemispherical cups and allograft [24]. (5) Primary stability of the press-fit cup was achieved in all cases in this series. Those hips which could not achieve primary stability by this technique were excluded. (6) All hips were performed by a single surgeon using a standard technique. All patients were compliant to the postoperative protocol with regular follow-ups. The good results of our patients might stem from appropriate patient selection, good surgical indications, and delicate grafting technique.

There is an increasing popularity in the use of modular porous metal augments to manage bone defect [36, 40]. However, the durability of these implants remains to be validated [1]. These highly porous metals have several advantages, including easier surgical technique, no risk of disease transmission and immunogenicity, and allowing rapid bone ingrowth [10, 11]. In addition, the porous surface of the augments provides high frictional force for immediate structural support [12]. However, a metal augment is safer to use mostly in elderly or less active patients with whom the requirement for bone stock restoration is lower [9, 13]. Moreover, catastrophic bone defect could result from removal of osseointegrated augments once deep sepsis occurs.

The study has limitations. First, it was a retrospective review and the case numbers were small. However, the clinical and radiological data of all patients were comprehensively recorded and analyzed. We could hardly find literature on this topic with large series, possibly reflecting the fact that incorporating the structural allograft into revision was technically demanding and its source was also limited. Second, the assessment of the acetabular defects, the percentage of allograft-host bone contact, and the decision of using uncemented hemispherical components were subjectively decided by the attending surgeon. Third, selection bias was inevitable in this study, because primary stability of the implant could be achieved in all cases in this study and those cases where primary implant stability could not be achieved were excluded from this study.

In conclusion, the current study showed that the use of morselized and structural allografts in combination with a cementless press-fitting cup could be a viable technique in acetabular revision. Delicate surgical technique, reasonable indications, successful bone stock restoration, and a securely fixed implant make this technique a favorable choice of revision for acetabular defect, particularly in young and active patients.

## Figures and Tables

**Table 1:** Causes for revision surgery.

	Number of cases	Percentage
Aseptic cup loosening	54	91.5%
Aseptic cage loosening	2	3.3%
Hemiarthroplasty acetabular erosion	2	3.3%
Septic loosening with infection control	1	1.9%

**Table 2:** Demographics and function score.

	Paprosky II	Paprosky IIIA	Paprosky IIIB
Gender (M/F)	6/4	8/13	10/18
Case number	10	21	28
Failure case number	0	1	2
Cup survival rate	100%	95.2%	92.8%
Follow-up (mean, year)	9.1	9.3	8.1
Harris Hip Score (range)			
Pre-op	68.5 (52.1–73.2)	66.3 (51.3–71.2)	52.4 (45.2–60.3)
Post-op	93.3 (87.5–98)	91.3 (82.3–95)	90.5 (80.1–93.2)
*p* value	<0.001	<0.001	<0.001

*p* values comparing preoperative and postoperative Harris Hip Score were determined using Student's *t*-test.
